# Infodemic in COVID-19 and its relationship with stress, depression, and anxiety in the elderly^1^

**DOI:** 10.1590/1518-8345.7769.4750

**Published:** 2026-03-30

**Authors:** Varinia Alejandra Rodríguez-Campo, Sandra Verónica Valenzuela-Suazo, Ricardo Bezerra-Cavalcante, Olivia Inés Sanhueza-Alvarado, Patricia del Tránsito Jara Concha, Aldo Vera-Calzaretta, Pablo Miguel Palma-Novoa

**Affiliations:** 1 Universidad de Concepción, Facultad de Enfermería, Concepción, Chile.; 2 Universidade Federal de Juiz de Fora, Faculdade de Enfermagem, Minas Gerais, MG, Brazil.; 3 Universidad de Atacama, Departamento Kinesiología, Atacama, Chile.; 4 Universidad de Concepción, Departamento Psiquiatría, Concepción, Chile.

**Keywords:** Infodemic, Psychological Stress, Depression, Anxiety, Aged, COVID-19.

## Abstract

**Objective:**

to analyze the phenomenon of the infodemic in COVID-19 and its relation with stress, depression, and anxiety in the elderly.

**Method:**

quantitative, correlational, cross-sectional study. Non-probabilistic sample of 195 elderly people, who answered a questionnaire for Infodemic, Perceived Stress Scale, Yesavage Geriatric Depression Scale, and Generalized Anxiety Scale through a telephone and/or online self-administered survey. Before you accept informed consent, descriptive and inferential statistics were used to analyze the data. The Ethics Committee approved the study.

**Results:**

the average age was 69 ± 6.2 years, and 58% were women. The average number of daily hours devoted to information was 5 ± 2.5, higher among men, with no significant differences between the sexes. Hours of infodemic were inversely related to mental health status, and the more hours of infodemic, the lower the level of stress, depression, and anxiety.

**Conclusion:**

the infodemic does not necessarily hurt the mental health of the elderly. From a nursing perspective, it is essential to strengthen critical thinking, promote digital literacy, and support networks and safe spaces that enhance autonomy and confront disinformation.

## Highlights

**(1)** Television was the medium most used by the elderly in Chile. **(2)** Seniors consumed an average of 5 daily hours of COVID-19 information. **(3)** TheThe number of dead and infected generated fear and anxiety among the elderly. **(4)** Many older people have experienced stress, depression, and anxiety during the pandemic. **(5)** The number of hours of information was inversely proportional to the level of stress, depression, and anxiety.

## Introduction

Humanity has constantly been exposed to various epidemics. The one caused by SARS-CoV-2 is undoubtedly one of the most complex experiences recently^1^, due to the high lethality and mortality caused by the virus in a short period^2^, whose epidemiological characteristics allowed it to spread rapidly from Wuhan, China, to the rest of the world^3^.

During this period, the population had to change its lifestyles to avoid the adverse effects of the virus. The World Health Organization (WHO) has advocated preventive measures such as hand washing, the use of personal protective equipment, social distancing, and confinement as one of many measures to contain the transmission of the pathogen^4^ ^-^ ^5^. The elderly were the age group most affected by the high mortality from COVID-19, due to the presence of comorbidities, geriatric syndromes, and the fragility of aging^6^.

The world is aging; 11% of the world’s population is made up of elderly people, and it is expected that by the 2050s this age group will have doubled, increasing the octogenarian and nonagenarian population^7^, which leads to an increase in age-related diseases, such as Alzheimer’s dementia and others, making it a group vulnerable to various diseases. This has made them a highly vulnerable group of people^8^ ^-^ ^10^, mainly due to the restrictive measures of confinement and social isolation, which have been considered harmful for the elderly population because they generate reduced physical activity, sleep disorders, cognitive impairment, and mental health problems such as fear and depression^8^ ^,^ ^11^.

In addition, during this pandemic period, millions of homes were inundated with a large volume of information transmitted through traditional and digital channels, informing people about the damage caused by COVID-19 and its therapeutic measures^8^. This information, which has been miscommunicated and disseminated as fake news on numerous occasions, lacks scientific backing and has generated misinformation among the public, leading them to make incorrect health decisions, as they contradicted treatment, pharmacological, and vaccine measures related to the pandemic^12^. Such was the magnitude of the information transmitted, often unfounded, that the WHO referred to it as “infodemics”, to describe information that is disseminated quickly and without further scientific evidence^13^ ^-^ ^14^.

Although restrictive measures were necessary to interrupt the chain of transmission of the virus, social distancing and confinement increased the problem of loneliness and isolation experienced by the elderly even before the pandemic^15^, and during this period there was a flood of alarming information about the virus, leading to feelings of fear, anxiety, uncertainty, stress, frustration, and other associated mental changes^8^ ^,^ ^15^.

Aging is a natural process that involves a series of anatomical, physiological, and psychological changes typical of this stage of life. In this context, mental health is crucial, as cognitive functioning in old age is a complex and individualized process that depends on personal resources, physical health, and accumulated life experiences. A higher prevalence of mental disorders and a high suicide rate among the elderly have been identified^16^, highlighting the urgent need to investigate this issue and the possible variables that can affect it, such as infodemic, to develop comprehensive strategies for the prevention and promotion of mental health. Currently, more than 15% of Chileans are elderly, as in the rest of the world^17^.

It is not known at a local level how the information received during the pandemic period has caused changes in their mental health, such as fear, anxiety, stress, and depression, so the study aims to analyze the phenomenon of the infodemic and its relation with stress, depression, and anxiety in the elderly.

## Method

### Type of study

A quantitative, non-experimental, cross-sectional, and correlational study that followed the recommendations of the Strengthening of Observational Studies in Epidemiology (STROBE).

### Location

The study was conducted in the Biobío region of Chile, comprising three provinces: Concepción, Biobío, and Arauco, each with its own communes.

### Period

Participants were recruited from March to September 2022.

### Population

Consists of all elderly people in the Biobío region, which for the year 2021 reached a total of N=261,502 elderly people.

### Selection criteria

The study included elderly individuals aged 60 or older who had a telephone line or email address and agreed to participate in the research. In addition, they had to be able to answer online or telephone surveys and use email and/or social media. Elderly people with cognitive impairment that prevented them from answering independently or who had difficulties understanding the study at the time of the interview were excluded.

### Sample definition

The non-probability sample was calculated using the formula, n = N.Z².p. (1-p) /Z².p. (1-p). + e². (N-1), using a sampling error of 5% and a confidence level of 95%, resulting in a sample of n=195 people.

### Study variables

The study included bio-sociodemographic variables (age, gender, marital status, income, etc.), variables related to the infodemic, measured through the number of daily hours of exposure to information about COVID-19 and the media used (television, radio, social networks), and mental health variables: stress, depression, and anxiety.

### Instruments used to collect information

An instrument created by the authors to collect bio-sociodemographic information and infodemics. The Perceived Stress Scale (PSS-14), the Geriatric Depression Scale (Yesavage), and the Generalized Anxiety Scale, all validated in Chile, were used to assess the mental health of the participants^18^ ^-^ ^20^.

The Perceived Stress Scale (PSS-14), developed by Cohen and adapted in Chile by Carvajal, et al.^19^ ^,^ ^21^, consists of 14 Likert-type questions with scores ranging from 0 to 4. The sum of the scores allows stress levels to be categorized: from 0 to 14 points indicates that stress never occurs or is seldom present; from 15 to 28 points, stress occurs occasionally; from 29 to 42 points, stress is frequent; and from 43 to 56 points, a high level of stress is present. The Abbreviated Geriatric Depression Scale, created by Sheikh and Yesavage^22^ and comprising 15 dichotomous questions, categorizes scores as follows: 0-5 points, normal; 6-10 points, mild depression; and 11-15 points, severe depression. On the other hand, the Geriatric Anxiety Inventory (IAG, its acronym in Spanish), created by Pachana^23^ and made up of 20 dichotomous questions, allows us to discriminate between people with or without anxiety, considering that a score equal to or greater than 8 indicates the presence of anxiety, and a score equal to or greater than 10 indicates generalized anxiety.

### Data collection

To collect the sample, the process began with the random selection of a telephone number from a list of elderly people in the Comprehensive Elderly Health Program in the Biobío region, Chile. Subsequently, the snowball method was used to complete the sample. The data were obtained through telephone surveys carried out by trained interviewers or self-administered, according to the participants’ preference, and were entered into a Google Forms form, ensuring no duplication of responses.

### Statistical analysis

The answers obtained through the Google Forms form were exported to the statistical packages JASP (version 0.15.0.0), Jamovi (version 2.3.18), and IBM SPSS Statistics (version 26.0) for analysis. Descriptive and inferential statistics were used in the study. Before conducting the inferential analysis, a normality test was performed on the variable “hours of infodemic”, which did not meet the normality criteria. Subsequently, the Kolmogorov-Smirnov test was applied, which showed significant values for the variables stress, depression, and anxiety. Considering these findings, it was decided to use the Kruskal-Wallis statistic, as nonparametric methods were deemed necessary. To further explore the differences between the groups, a *post hoc* analysis was carried out using the Dwass-Steel-Critchlow-Fligner (DSCF) test. This test is beneficial for comparing several groups in situations where significant differences have been identified in a general analysis, allowing to determine specifically which groups show statistically significant differences between each other. The dataset supporting the results of this study is available in Mendeley Data, V1, at https://doi.org/10.17632/xysrhct5ny.1.

### Ethical considerations

This study was based on Ezequiel Emanuel’s eight requirements, as outlined in the standards of the Council for International Organizations of Medical Sciences (CIOMS) and the Declaration of Helsinki^24^. Participants had to provide informed consent, in which the researcher guaranteed the confidentiality of their answers, as well as informing them of the risks and benefits associated with the study. Acceptance of consent was recorded through confirmation on the web survey. In the case of telephone surveys, the reading of the consent and its acceptance were recorded, ensuring storage in an external memory exclusively for the study, in accordance with Chilean legislation applicable to research involving human beings. The final document, signed by the principal investigator, was sent to each participant via email or authorized social network. This study was approved by the Ethics Committee of the Faculty of Nursing, by the Ethics Committee of the Vice-Rectorate of Research and Development of the University of Concepción, and by the accredited Ethics Committee of the Concepción Health Service, with the code CEC-SSC:21-08-44 of the year 2021.

## Results

The descriptive analysis, presented in Table 1, reveals that the sample studied had an average age of 69 years, with a standard deviation of ±6.2 years, comprising mainly women, most of whom were married, and a low percentage of whom lived alone. It is worth noting that most participants are retired, and 65% receive a monthly income of 300,000 Chilean *pesos* (approximately equivalent to 300 US dollars).

**Table 1:** Biosociodemographic profile of the elderly in the study. Biobío Region, Chile, 2022

Variables	Categories	Fr*	%^†^
Gender	Female	113	58%
	Male	82	42%
Marital status	Married	90	46.1%
	Widowed	52	26.7%
	Single	18	9.2%
	Living together	18	9.2%
	Separated	10	5.1%
	Divorced	7	3.6%
Income source	Retired	175	89.7%
	Employed	15	7.7%
	Government benefit	1	1%
Monthly income	< 300 US$^‡^	127	65.1%
	301-500 US$^‡^	37	18.9%
	501-850 US$^‡^	15	7.8%
	851-1500 US$^‡^	3	1.5%
	> 1501 US$^‡^	3	1.5%
	Did not answer	10	5.1%
Change in income due to the pandemic	Decreases	32	16.4%
	Increases	31	15.8%
	Does not vary	121	62%
	Did not answer	11	5.6%

*FR = Frequency; ^†^% = Percentage; ^‡^US dollar = 917 Chilean *pesos*, 09/30/2022

Of the elderly people studied, 96% reported being exposed to information broadcast on television, 69% on social media, and 68% on the radio. Concerning the impact generated by the information disseminated during the pandemic, 75% of the elderly reported feeling physical and psychological effects from the information disseminated on television, 49% from the information communicated on the radio, and 52% felt affected by the viralization of content on social networks, mainly Facebook and WhatsApp. Among the types of information received, the one that generated the most fear among the participants was related to the number of dead and infected, with the figures broadcast on television being particularly alarming, as stated by 63% of the elderly.

The total time spent exposed to information was calculated by adding up the hours spent on television, radio, and social networks, yielding an average of 5.0 hours (± 2.5 hours), with a variation ranging from 0 to 12 hours. When this data was broken down by gender, it was observed that men received information for an average of 5.2 hours (± 2.1 hours), while women received it for 4.9 hours (± 2.7 hours), which shows a difference of 0.3 hours. However, this difference was not statistically significant, with a p-value of 0.204, suggesting that the time of exposure to information does not differ significantly between the two genders.

Table 2 summarizes the levels of stress, depression, and anxiety experienced by the elderly participants, with the respective daily hours spent receiving information about COVID-19 during the pandemic. For each of the levels mentioned above, the average number of hours that participants dedicated to information related to the pandemic is indicated. 

**Table 2:** Daily hours of exposure to information about COVID-19 according to the level of stress, depression, and anxiety in the elderly. Biobío Region, Chile, 2022

Hours of information						
Scales	Level	Range*	Fr^†^	%^‡^	Mean^§^	SD^||^
Perceived stress (PSS-14)	Not detected	0-14	67	34%	5.5	2.4
	Occasionally	15-28	114	58%	5.0	2.4
	Often	29-42	14	7%	3.0	2.5
	Very stressed	43-56	0	0%	0	0
Depression (Yesavage)	Normal	0-5	143	73%	5.4	2.5
	Mild depression	06-10	35	18%	4.6	2.0
	Severe depression	11-15	17	9%	3.5	2.3
Geriatric Anxiety Inventory (GAI)	Not detected	0-7	148	76%	5.5	2.4
	Possible anxiety	08-09	10	5%	4.6	2
	Generalized anxiety	10-20	37	19%	3.5	2.4

*Range = Scale score range; ^†^Fr = Frequency; ^‡^% = Percentage; ^§^Average = Mean; ^||^SD = Standard Deviation

Table 3 summarizes the results of the Kruskal-Wallis test for the variables hours of infodemic and presence of stress, depression, and anxiety in the elderly.

The results indicate that there are significant differences between the hours of information received and the different mental illnesses. The conclusions of this *post hoc* analysis are summarized in Table 4.

**Table 3:** Relationship between hours of COVID-19 information and the presence of mental health disorders in the elderly. Biobío Region, Chile, 2022

Variables	χ²*	df^†^	p^‡^	ε²^§^
Information hours/perceived stress	12.2	2	0.002^||^	0.061
Hours of information/depression	12.4	2	0.002^||^	0.062
Hours of information/anxiety	20.7	2	<.001^||^	0.104

*χ² = Chi-squared statistics; ^†^df = Degrees of freedom; ^‡^p = Level of significance; ^§^ **ε²** = Effect size; ^||^Significance <0.01

**Table 4:** Steel-Dwass-Critchlow-Fligner results for hours of infodemic, stress, depression and anxiety in the elderly. Biobío Region, Chile, 2022

Scales	Gr 1*	Gr 2^†^	W^‡^	p^§^
Stress level	Not detected	Occasionally	-1.55	0.516
	Not detected	Frequently	-4.86	0.002^ǁ^
	Occasionally	Frequently	-4.29	0.007^¶^
	Normal	Mild	-2.61	0.154
Level of depression	Normal	Severe	-4.50	0.004^ǁ^
	Mild	Severe	-2.72	0.133
	Not detected	Possible anxiety	-1.55	0.516
Level of anxiety	Not detected	Generalized anxiety	-6.36	<.001^¶^
	Possible anxiety	Generalized anxiety	-2.13	0.287

*Gr 1 = Group 1; ^†^Gr 2 = Group 2; ^‡^W = Comparison of groups; ^§^p = Significance level; ^ǁ^Significance p<0.05; ^¶^Significance p< 0.01

Table 4 shows that there are significant differences between the groups in terms of the different levels of stress, depression, and anxiety studied.

When analyzing and comparing the results presented in Tables 1 and 3 on the stress scale, the group that did not experience stress had an average exposure to information that was two and a half hours longer compared to the group that experienced stress “frequently”. A similar situation is observed between the “occasionally” and “frequently” groups on the same scale. 

About the depression scale, significant differences were also identified between the groups, with a difference of two hours of exposure between the group classified as usual and the group showing a severe level of depression. Similarly, on the generalized anxiety scale, the elderly who did not show symptoms of anxiety had two hours more exposure to the information compared to the group that showed signs of generalized anxiety. It is interesting to note that the groups who did not report stress, depression, or anxiety had, on average, greater exposure to information compared to those who experienced these problems more frequently. 

## Discussion

This study revealed a relationship between infodemic and mental health in Chile’s elderly population. When comparing these results with global trends, significant differences are evident in relation to other international studies. While in the global context, infodemic showed a positive and more consistent association with the mental disorders analyzed, in Chile, the relationship appears to be less pronounced^25^ ^-^ ^27^. These results suggest that there are probably other factors, such as cultural, social, or economic factors specific to the Chilean population, that may influence how infodemic affects the mental health of the elderly. This discrepancy underscores the importance of considering the local context when assessing the impact of the infodemic on mental health, and it paves the way for future research that examines the specificities of the Chilean experience in comparison with other regions of the world.

During the pandemic, the elderly were affected by isolation and social distancing, which left them feeling entirely alone^4^ ^,^ ^28^. This period favored devoting time to information, which was preferably consumed through traditional means, as reported by the elderly in this study. The loneliness imposed by isolation and the lack of communication prevalent during this period gave way to a focus on the countless hours of communication about the pandemic.

Unlike other international studies, in Chile, the elderly showed that more hours of exposure to information were associated with a lower incidence of mental health problems. This result undoubtedly prompts the authors to reflect on the phenomenon under study. The apparent paradox, in comparison with international findings, raises the debate whether individuals who showed symptoms of stress, depression, and anxiety could have been more sensitive to the information they received. It has been shown that high levels of stress, depression, and anxiety decrease the ability to process and retain information; in addition, these mental alterations can negatively affect cognitive function, which can hinder a person’s ability to concentrate and retain information^17^ ^,^ ^29^ ^-^ ^30^. On the other hand, those who did not show symptoms of stress, depression, or anxiety may have been better able to handle and process the information, which allowed them to maintain a higher level of exposure without being negatively affected by the information received.

Likewise, it is essential to consider the relevance of television, which, as a massive and accessible source of information in Chile for the individuals studied, may have provided reliable and valuable content during the pandemic, unlike international studies in which the most significant information consumption occurred on social networks^26^. In this sense, television could become a preventative medium, helping the elderly to stay properly informed and better manage their emotions in the face of new critical events. Thus, the quality and credibility of the information received play a crucial role in how individuals perceive and respond to mental health challenges.

It is essential to point out that, despite the positive effect observed in this group of elderly people in relation to the infodemic, this age group experienced fear and anxiety when faced with information about the number of dead and infected during this period. Many participants manifested physical and psychological ailments, which is consistent with the results of other international studies^31^. This contradiction suggests that, although exposure to information may have had protective effects in some respects, the negative emotional impact associated with alarming figures may have generated a significant response of fear and anxiety in the elderly population.

Indeed, during the COVID-19 pandemic, the elderly have become a highly vulnerable group to the adverse effects of the virus. This was evident from the large number of people infected and subsequently killed by the disease^8^. Unfortunately, health problems have not been limited to physical conditions; they have also manifested themselves significantly around mental health^32^. Before the pandemic, the mental health of the elderly was already a significant health problem. Previous studies have indicated that almost 20% of this age group suffers from some mental health problems, with varying degrees of intensity, which in turn are related to cognitive issues. Thus, depression has a substantial relationship with cognitive functions such as language, memory, attention, and executive function, among others^30^, and perhaps this characteristic is related to the findings of this study.

Undoubtedly, the infodemic, a phenomenon declared by the WHO as a communicative pandemic, represents a significant challenge for health professionals, especially nursing professionals. They can play a crucial role in combating the infodemic by providing accurate and up-to-date information to the elderly, which will help them better understand the situation and make informed decisions about their health^34^. It is essential to provide health education on how to cope with complex conditions, such as the pandemic, including practical advice on managing stress and anxiety^35^. Additionally, it is crucial to provide emotional support to the elderly, enabling them to manage their emotions and alleviate stress and anxiety in the face of complex health issues. This comprehensive approach not only improves the psychological well-being of this age group but also contributes to their ability to cope with the challenges arising from the infodemic.

However, one of the limitations of this study was the date the data was obtained, which may have influenced the perception and/or recollection of the phenomenon. The circumstances and nature of the information available at the time may have affected how the participants interpreted and reacted to the infodemic and its relationship with mental health. This temporal factor is crucial for understanding the dynamics of perception, emotional responses in crises, and recall.

## Conclusion

The study concludes that the relationship between exposure to information and mental health in the elderly was not linear at the local level. Therefore, greater exposure does not always imply a negative impact on mental health. However, it is interesting to study the coping strategies that older people use to deal with the infodemic. From a nursing perspective, it is essential to implement strategies that promote mental health to cope with misinformation, such as enhancing digital and health literacy, continuing education in the responsible use of media, fostering emotional coping skills, and creating safe spaces for active listening and emotional expression. In addition, it is essential to promote community support networks that strengthen the social and emotional support of the elderly, as well as psycho-educational interventions that boost their autonomy, self-esteem, and critical capacity in the face of information.

## Data Availability

The dataset of this article is available on the RLAE page in the Mendeley Data repository, at the link DOI: https://doi.org/10.17632/xysrhct5ny.1
