# Supplementary material for: Infodemic in COVID-19 and its relationship with stress, depression, and anxiety in the elderly
Source: Rev Lat Am Enfermagem. 2026 Mar 30;34:e4750. doi: 10.1590/1518-8345.7769.4750 (PMC13035207; doi:10.1590/1518-8345.7769.4750)
Supplement: Translation

# Translation: Infodemia na COVID-19 e sua relação com estresse, depressão e ansiedade em idosos^2^

**DOI:** 10.1590/1518-8345.7769.4751

**Authors:** Varinia Alejandra Rodríguez-Campo, Sandra Verónica Valenzuela-Suazo, Ricardo Bezerra-Cavalcante, Olivia Inés Sanhueza-Alvarado, Patricia del Tránsito Jara Concha, Aldo Vera-Calzaretta, Pablo Miguel Palma-Novoa

**Affiliations:** 1 Universidad de Concepción, Facultad de Enfermería, Concepción, Chile.; 2 Universidade Federal de Juiz de Fora, Faculdade de Enfermagem, Minas Gerais, MG, Brasil.; 3 Universidad de Atacama, Departamento Kinesiología, Atacama, Chile.; 4 Universidad de Concepción, Departamento Psiquiatría, Concepción, Chile.

**Keywords:** Infodemia, Estresse Psicológico, Depressão, Ansiedade, Idoso, COVID-19.

## Abstract

**Objetivo:**

analisar o fenômeno da infodemia na COVID-19 e sua relação com estresse, depressão e ansiedade em idosos.

**Método:**

estudo quantitativo, correlacional, transversal. Amostra não probabilística de 195 idosos, que responderam a um questionário para: Infodemia, Escala de Estresse Percebido, Escala de Depressão Geriátrica de Yesavage, Escala de Ansiedade Generalizada, por meio de inquérito telefônico e/ou autoaplicável *online*. Aceitação prévia do consentimento informado. Para análise dos dados, utilizou-se estatística descritiva e inferencial. O estudo foi aprovado pelo Comitê de Ética.

**Resultados:**

a média de idade foi de 69 ± 6,2 anos, 58% eram mulheres. O número médio de horas diárias destinadas à informação foi de 5±2,5, sendo maior nos homens, sem diferenças significativas entre os sexos. As horas de infodemia foram inversamente relacionadas à condição de saúde mental, assim como, quanto maior a hora de informação, menor o nível de estresse, depressão e ansiedade.

**Conclusão:**

a infodemia não tem necessariamente um impacto negativo na saúde mental dos idosos. A partir da enfermagem, é fundamental fortalecer o pensamento crítico, o letramento digital, promover redes de apoio e espaços seguros que potencializem a autonomia e o enfrentamento da desinformação.

## Destaques

**(1)** A televisão foi o meio de comunicação mais utilizado pelos idosos no Chile. **(2)** Uma média de 5 horas diárias de informações sobre COVID-19 foi consumida por idosos. **(3)** O número de mortos e infectados gerou medo e ansiedade nos idosos. **(4)** Muitos idosos experimentaram estresse, depressão e ansiedade durante a pandemia. **(5)** O número de horas de informações foi inversamente proporcional ao nível de estresse, depressão e ansiedade.

## Introdução

A humanidade sempre esteve exposta a várias epidemias. Sem dúvida, aquela causada pelo Sars-Cov 2 foi uma das mais complexas experimentadas nos últimos anos^1^, devido à alta letalidade e mortalidade causada pelo vírus em um curto período^2^, cujas características epidemiológicas permitiram sua rápida disseminação de Wuhan, China, para o resto do mundo^3^.

Durante esse período, a população como um todo teve que mudar seus estilos de vida para evitar os efeitos adversos do vírus. A Organização Mundial da Saúde (OMS) preconizou medidas preventivas, como a lavagem das mãos, o uso de elementos de proteção individual, o distanciamento social e o confinamento como algumas das muitas medidas para conter a transmissão do patógeno^4^ ^-^ ^5^, principalmente entre idosos, que foram a faixa etária mais comprometida devido à alta mortalidade por COVID-19, em função da presença de comorbidades, síndromes geriátricas e fragilidade do envelhecimento^6^.

O mundo está envelhecendo, 11% da população mundial corresponde a pessoas idosas. Espera-se que até a década de 2050 essa faixa etária tenha dobrado, aumentando a população octo e nonagenária^7^, o que acarreta um aumento de doenças relacionadas à idade, como demência de Alzheimer e outras, tornando-se este um grupo altamente vulnerável a várias doenças, maus-tratos e desastres^7^ ^-^ ^10^. As medidas restritivas de confinamento e isolamento social foram consideradas negativas para a população idosa por gerarem redução da atividade física, distúrbios do sono, comprometimento cognitivo e problemas de saúde mental, como medo e depressão^8^ ^,^ ^11^.

Além disso, durante esse período de pandemia, milhões de lares foram inundados com um grande volume de informações que foram transmitidas por canais tradicionais e informatizados, comunicando às pessoas sobre os prejuízos gerados pela COVID-19 e suas medidas terapêuticas^8^. Essas informações em muitas ocasiões foram comunicadas de forma incorreta e até mesmo como *fake news*, sem respaldo científico, gerando desinformação nas pessoas, orientando-as a tomar decisões erradas em saúde, pois contrariaram medidas de tratamento, farmacológicas e vacinais relacionadas à pandemia^12^. Tamanha foi a magnitude das informações transmitidas, muitas vezes infundadas, que a OMS as chamou de “infodemia”, para se referir a informações que são divulgadas rapidamente e sem maiores evidências científicas^13^ ^-^ ^14^.

Embora medidas restritivas tenham sido necessárias para interromper a cadeia de transmissão do vírus, o distanciamento social e o confinamento aumentaram o problema da solidão e do isolamento que os idosos apresentavam, mesmo antes da pandemia^15^, e, ainda, durante esse período houve uma enxurrada de informações alarmantes sobre o vírus, levando a sentimentos de medo, ansiedade, incerteza, estresse, frustração e outras alterações mentais associadas^8^ ^,^ ^15^.

O envelhecimento é um processo natural que envolve uma série de alterações anatômicas, fisiológicas e psicológicas típicas dessa fase da vida. Nesse contexto, a saúde mental adquire particular relevância, uma vez que o funcionamento cognitivo na velhice é um processo individual que depende de recursos pessoais, estado de saúde física e experiências de vida acumuladas. Identificou-se maior prevalência de transtornos mentais e alta taxa de suicídio entre idosos^16^, destacando a necessidade urgente de investigar esse tema e as possíveis variáveis que podem afetá-lo, como a infodemia, a fim de desenvolver estratégias abrangentes para a prevenção e promoção da saúde mental. Atualmente, mais de 15% dos chilenos são idosos, semelhante ao resto do mundo^17^.

Não se conhece, a nível local, como as informações recebidas durante o período pandêmico provocaram alterações na sua saúde mental, como medo, ansiedade, estresse e depressão, pelo que o objetivo do estudo é analisar o fenômeno da infodemia e sua relação com com estresse, depressão e ansiedade em idosos.

## Método

### Tipo de estudo

Estudo quantitativo, não experimental, transversal e correlacional que seguiu as recomendações do *Strengthening the Reporting of Observational Studies in Epidemiology* (STROBE).

### Local

O estudo foi realizado na região de Biobío, Chile, composta por três províncias: Concepción, Biobío e Arauco, cada uma com suas respectivas comunas.

### Período

O recrutamento dos participantes foi realizado durante o período de março a setembro de 2022.

### População

Constituída por todos os idosos da região de Biobío, o que, para o ano de 2021, atingiu um total de N=261.502 idosos.

### Critérios de seleção

Foram incluídos no estudo idosos com 60 anos ou mais, que possuíssem linha telefônica ou e-mail e que concordassem em participar da pesquisa. Além disso, eles deveriam ter capacidade de responder a pesquisas *online* ou por telefone e usar e-mail e/ou mídia social. Foram excluídos os idosos com comprometimento cognitivo que os impedisse de responder de forma autônoma ou que apresentassem dificuldades de compreensão da pesquisa no momento da entrevista.

### Definição da amostra

A amostra não probabilística foi calculada com base na fórmula n = N.Z².p. (1-p) /Z².p. (1-p). + e². (N-1), utilizando-se um erro amostral de 5% e um nível de confiança de 95%, o que resultou numa amostra de n=195 pessoas.

### Variáveis do estudo

Foram incluídas variáveis biossociodemográficas (idade, sexo, estado civil, situação conjugal, renda etc.), variáveis relacionadas à infodemia, medidas por meio do número de horas diárias de exposição a informações sobre a COVID-19 e meios de comunicação utilizados (televisão, rádio, redes sociais), e variáveis de saúde mental: estresse, depressão e ansiedade.

### Instrumentos utilizados para recolher informações

Utilizou-se instrumento criado pelos autores para coletar informações biossociodemográficas e infodemias. Para avaliar a saúde mental dos participantes, foram utilizados: Escala de Estresse Percebido (PSS-14), Escala de Depressão Geriátrica (Yesavage) e Inventário de Ansiedade Generalizada (GAI), todos validados no Chile^18^ ^-^ ^20^.

A Escala de Estresse Percebido (PSS-14), criada por Cohen e validada no Chile por Carvajal, et al.^19^ ^,^ ^21^, é composta por 14 questões do tipo Likert, com escores que variam de 0 a 4. A soma dos pontos permite categorizar os níveis de estresse: de 0 a 14 pontos indica que o estresse nunca ou quase nunca ocorre; de 15 a 28 pontos, estresse ocasional; de 29 a 42 pontos, estresse frequente; e de 43 a 56 pontos, um alto nível de estresse. A Escala Abreviada de Depressão Geriátrica, criada por Sheikh e Yesavage^22^ ^)^ e composta por 15 questões dicotômicas, categoriza os escores em: de 0 a 5 pontos, normal; de 6 a 10 pontos, depressão leve; e de 11 a 15 pontos, depressão grave. Por outro lado, o Inventário de Ansiedade Geriátrica (IAG), criado por Pachana^23^ e composto por 20 questões dicotômicas, permite discriminar pessoas com ou sem ansiedade, considerando que um escore igual ou maior que 8 indica a presença de ansiedade e um escore igual ou maior que 10 indica ansiedade generalizada.

### Coleta de dados

Para a coleta da amostra, iniciou-se o processo com a seleção aleatória de um número de telefone de uma lista de idosos do Programa de Saúde Integral do Idoso, na região de Biobío, Chile. Posteriormente, o método bola de neve foi utilizado para completar a amostra. Os dados obtidos por meio de inquéritos telefônicos realizados por entrevistadores treinados ou autoaplicáveis, de acordo com a preferência dos participantes, foram digitados em um formulário do *Google Forms*, garantindo-se a não duplicidade de respostas.

### Análise estatística

As respostas obtidas por meio do formulário do *Google Forms* foram exportadas para os pacotes estatísticos JASP (versão 0.15.0.0), Jamovi (2.3.18) e IBM SPSS *Statistics* (26.0) para análise. Estatísticas descritivas e inferenciais foram utilizadas na análise. Previamente à análise inferencial, foi realizado um teste de normalidade na variável horas de infodemia, que não atendeu aos critérios de normalidade. Posteriormente, foi aplicado o teste de Kolmogorov-Smirnov, que mostrou valores significativos para as variáveis estresse, depressão e ansiedade. Diante desses achados, optou-se por utilizar a estatística de Kruskal-Wallis, baseando-se na necessidade de aplicação de métodos não paramétricos. Para explorar ainda mais as diferenças entre os grupos, foi realizada uma análise *post hoc* usando o teste de Dwass-Steel-Critchlow-Fligner (DSCF). Este teste é especialmente útil para comparar vários grupos em situações em que foram identificadas diferenças significativas numa análise geral, permitindo determinar especificamente quais os grupos que apresentam diferenças estatisticamente significativas entre si. O conjunto de dados que sustenta os resultados deste estudo está disponível em *Mendeley Data*, V1, por meio do link: https://doi.org/10.17632/xysrhct5ny.1.

### Considerações éticas

Este estudo fundamentou-se nos oito requisitos de Ezequiel Emanuel, com base nas normas do *Council of International Organizations of Medical Sciences* (CIOMS) e na Declaração de Helsinki^24^. Os participantes tiveram que fornecer consentimento informado, no qual o pesquisador garantiu o sigilo das respostas, bem como a menção de riscos e benefícios associados ao estudo. A aceitação do Termo de Consentimento foi registrada por confirmação na pesquisa na web e, no caso de pesquisas telefônicas, a leitura do consentimento e sua aceitação foram registradas, garantindo o armazenamento em memória externa exclusiva para o estudo, de acordo com a legislação chilena aplicável à pesquisa com seres humanos. O documento final, assinado pelo pesquisador principal, foi enviado a cada participante em seu e-mail ou rede social autorizada. Este estudo foi aprovado pelo Comitê de Ética da Faculdade de Enfermagem, pelo Comitê de Ética da Vice-Reitoria de Pesquisa e Desenvolvimento da *Universidad de Concepción* e pelo Comitê de Ética credenciado do Serviço de Saúde de Concepción, com o código CEC-SSC:21-08-44 do ano de 2021.

## Resultados

A análise descritiva, apresentada na Tabela 1, revela que a amostra estudada apresentou uma idade média de 69 anos, com um desvio-padrão de ± 6,2 anos, sendo composta principalmente por mulheres, a maioria casadas com baixa porcentagem vivendo sozinhas. É importante destacar que a maioria dos participantes é aposentada e 65% recebem uma renda mensal de 300 mil pesos chilenos (equivalente a aproximadamente 300 dólares americanos).

**Tabela 1:** Perfil biossociodemográfico de idosos do estudo. Região de Biobío, Chile, 2022

Variáveis	Categorias	Fr*	%^†^
Sexo	Feminino	113	58%
	Masculino	82	42%
Estado civil	Casado/a	90	46,1%
	Viúvo/a	52	26,7%
	Solteiro/a	18	9,2%
	Convivência	18	9,2%
	Separado/a	10	5,1%
	Divorciado/a	7	3,6%
Fonte de renda	Aposentadoria	175	89,7%
	Empregado/a	15	7,7%
	Benefício Governamental	1	1%
Renda mensal	< 300 US$^‡^	127	65,1%
	301-500 US$^‡^	37	18,9%
	501-850 US$^‡^	15	7,8%
	851-1500 US	3	1,5%
	> 1501 US$^‡^	3	1,5%
	Não respondeu	10	5,1%
Variação na renda devido à pandemia	Diminui	32	16,4%
	Aumenta	31	15,8%
	Não varia	121	62%
	Não respondeu	11	5,6%

*FR = Frequência; ^†^% = Porcentagem; ^‡^Dólar estadounidense = 917 pesos chilenos, 30/09/2022

Dos idosos estudados, 96% declararam ter sido expostos às informações veiculadas pela televisão, 69% às redes sociais e 68% ao rádio. Quanto ao impacto gerado pelas informações divulgadas durante a pandemia, 75% dos idosos relataram sentir um impacto físico e psicológico pelas informações divulgadas pela televisão, 49% pelas informações divulgadas pelo rádio e 52% se sentiram afetados pela viralização de conteúdo nas redes sociais, principalmente *Facebook* e *WhatsApp*. Entre os tipos de informação recebida, a que mais gerou medo entre os participantes foi aquela relacionada ao número de mortos e infectados, sendo particularmente alarmantes os números divulgados pela televisão, mencionados por 63% dos idosos.

O tempo total de exposição à informação foi calculado somando as horas dedicadas à televisão, ao rádio e às redes sociais, obtendo-se uma média de 5,0 horas (± 2,5 horas) diárias, com uma variação entre 0 e 12 horas. Ao detalhar esses dados por sexo, observou-se que os homens receberam informações durante uma média de 5,2 horas (± 2,1 horas), enquanto as mulheres receberam durante 4,9 horas (± 2,7 horas), o que evidencia uma diferença de 0,3 horas. No entanto, essa diferença não foi estatisticamente significativa, com um valor de p = 0,204, o que sugere que o tempo de exposição à informação não difere de maneira relevante entre os dois sexos.

A Tabela 2 resume os níveis de estresse, depressão e ansiedade experimentados pelos idosos participantes, com as respectivas horas diárias dedicadas à recepção de informações sobre a COVID-19 durante a pandemia. Para cada um dos níveis mencionados, são indicadas as horas médias que os participantes dedicaram às informações relacionadas à pandemia.

**Tabela 2:** Horas diárias de exposição a informações sobre a COVID-19 conforme o nível de estresse, depressão e ansiedade em idosos. Região de Biobío, Chile, 2022

Horas de informação						
Escalas	Nível	Intervalo*	Fr^†^	%^‡^	Média^§^	DP^||^
Estresse percebido (PSS-14)	Não detectado	0-14	67	34%	5,5	2,4
	Ocasionalmente	15-28	114	58%	5,0	2,4
	Frequentemente	29-42	14	7%	3,0	2,5
	Muito estressado	43-56	0	0%	0	0
Depressão (Yesavage)	Normal	0-5	143	73%	5,4	2,5
	Depressão leve	06-10	35	18%	4,6	2,0
	Depressão grave	11-15	17	9%	3,5	2,3
Inventário de Ansiedade Generalizada (GAI)	Não detectada	0-7	148	76%	5,5	2,4
	Possível ansiedade	08-09	10	5%	4,6	2
	Ansiedade generalizada	10-20	37	19%	3,5	2,4

*Intervalo = Intervalo de pontuação da escala; ^†^Fr = Frequência; ^‡^% = Porcentagem; ^§^Média = Média; ^||^DP = Desvio-padrão

A Tabela 3 resume os resultados do teste de Kruskall-Wallis para as variáveis horas de infodemia e presença de estresse, depressão e ansiedade em idosos.

Os resultados revelam a existência de diferenças significativas entre as horas de informação recebida e as diferentes doenças mentais. As conclusões desta análise *post hoc* estão resumidas na Tabela 4.

Ao observar a Tabela 4, é possível evidenciar que existem diferenças significativas entre os grupos dos diferentes níveis de estresse, depressão e ansiedade estudados.

**Tabela 3:** Relação entre horas de informação de COVID-19 e a presença de alterações de saúde mental em idosos. Região de Biobío, Chile, 2022

Variáveis	χ²*	df^†^	p^‡^	ε²^§^
Horas de informação/Estresse percebido	12,2	2	0,002^||^	0,061
Horas de informação/Depressão	12,4	2	0,002^||^	0,062
Horas de informação/Ansiedade	20,7	2	<,001^||^	0,104

*χ² **=** Estatística de Qui-quadrado; ^†^df = Graus de liberdade; ^‡^p = Nível de significância; ^§^ **ε²** = Tamanho do efeito; ^||^Significância <0,01

**Tabela 4:** Resultados de Steel-Dwass-Critchlow-Fligner para horas de infodemia, estresse, depressão e ansiedade de idosos. Região de Biobío, Chile, 2022

Escalas	Gr 1*	Gr 2^†^	W^‡^	p^§^
Nível de estresse	Não detectado	Ocasionalmente	-1,55	0,516
	Não detectado	Frequentemente	-4,86	0,002^ǁ^
	Ocasionalmente	Frequentemente	-4,29	0,007^¶^
	Normal	Leve	-2,61	0,154
Nível de depressão	Normal	Grave	-4,50	0,004^ǁ^
	Leve	Grave	-2,72	0,133
	Não detectada	Possível ansiedade	-1,55	0,516
Nível de ansiedade	Não detectada	Ansiedade generalizada	-6,36	<,001^¶^
	Possível ansiedade	Ansiedade generalizada	-2,13	0,287

*Gr 1 = Grupo 1; ^†^Gr 2 = Grupo 2; ^‡^W = Comparação de grupos; ^§^p = Nível de significância; ^ǁ^Significância p<0,05; ^¶^Significância p< 0,01

Ao analisar e comparar os resultados apresentados nas Tabelas 1 e 3, é possível observar que, na escala de estresse, o grupo que não apresentou estresse teve uma exposição média à informação de duas horas e meia a mais em comparação com o grupo que experimentou estresse “frequentemente”. Uma situação semelhante é observada entre os grupos “ocasionalmente” e “frequentemente” na mesma escala.

No que diz respeito à escala de depressão, também foram identificadas diferenças significativas entre os grupos, destacando-se uma diferença de duas horas de exposição entre o grupo classificado como normal e o grupo que apresenta um nível grave de depressão. Da mesma forma, na escala de ansiedade generalizada, verifica-se que os idosos que não apresentaram sintomas de ansiedade tiveram duas horas a mais de exposição à informação em comparação com o grupo que apresentou sinais de ansiedade generalizada. É interessante observar que os grupos que não relataram estresse, depressão ou ansiedade tiveram, em média, uma maior exposição à informação em comparação com aqueles que experimentaram esses problemas com maior frequência.

## Discussão

Este estudo revelou uma relação entre a infodemia e a saúde mental na população idosa do Chile. Ao comparar esses resultados com as tendências observadas globalmente, destacam-se diferenças significativas em relação a outros estudos internacionais. Enquanto no contexto global a infodemia demonstrou uma associação positiva e mais consistente com os transtornos mentais analisados, no Chile a relação parece ser menos pronunciada^25^ ^-^ ^27^. Esses resultados sugerem que provavelmente existem outros fatores, tais como culturais, sociais ou econômicos, específicos da população chilena, que podem influenciar como a infodemia afeta a saúde mental dos idosos. Essa discrepância ressalta a importância de se considerar o contexto local ao avaliar o impacto da infodemia na saúde mental e abre caminho para futuras pesquisas que explorem as particularidades da experiência chilena em comparação com outras regiões do mundo.

Durante a pandemia, os idosos foram afetados pelo isolamento e distanciamento social, que os levou a permanecer completamente sozinhos^4^ ^,^ ^28^. Esse período favoreceu a dedicação de tempo à informação, que foi consumida preferencialmente por meios tradicionais, conforme relatado pelos idosos deste estudo. A solidão imposta pelo isolamento e a falta de comunicação predominantes nesse período deram lugar à atenção a inúmeras horas de comunicação sobre a pandemia.

Ao contrário de outros estudos internacionais, no Chile os idosos demonstraram que um maior número de horas de exposição à informação estava associado a uma menor incidência de alterações na saúde mental. Este resultado sem dúvida leva os autores a refletir sobre o fenômeno estudado. O aparente paradoxo, em comparação com as descobertas internacionais, suscita o debate sobre se os indivíduos que apresentaram sintomas de estresse, depressão e ansiedade poderiam ter sido mais sensíveis às informações recebidas. Demonstrou-se que altos níveis de estresse, depressão e ansiedade diminuem a capacidade de processar e reter informações, além disso, essas alterações mentais podem afetar negativamente a função cognitiva, o que pode dificultar a capacidade de concentração e retenção de informações^17^ ^,^ ^29^ ^-^ ^30^. Por outro lado, aqueles que não apresentaram sintomas de estresse, depressão ou ansiedade podem ter sido mais capazes de lidar e processar as informações, o que lhes permitiu manter um nível de exposição mais alto sem serem afetados negativamente pelas informações recebidas.

Da mesma forma, é importante considerar a relevância da televisão, que, por ser uma fonte de informação massiva e acessível no Chile para os indivíduos estudados, pode ter fornecido conteúdo confiável e útil durante a pandemia, ao contrário de estudos internacionais em que o maior consumo de conteúdo ocorreu nas redes sociais^26^. Nesse sentido, a televisão poderia se tornar um meio preventivo, ajudando os idosos a se manterem adequadamente informados e a gerenciar melhor suas emoções diante de novos eventos críticos. Assim, a qualidade e a credibilidade das informações recebidas desempenham um papel crucial na forma como os indivíduos percebem e respondem aos desafios da saúde mental.

É importante destacar que, apesar do efeito positivo observado nesse grupo de idosos em relação à infodemia, essa faixa etária experimentou medo e ansiedade diante das informações sobre o número de mortos e infectados durante esse período. Muitos participantes manifestaram afeções físicas e psicológicas, o que é consistente com os resultados de outros estudos internacionais^31^. Essa contradição sugere que, embora a exposição à informação possa ter tido efeitos protetores em alguns aspectos, o impacto emocional negativo associado a números alarmantes pode ter gerado uma resposta significativa de medo e ansiedade na população idosa.

Certamente, durante o período da pandemia da COVID-19, os idosos se tornaram um grupo altamente vulnerável aos efeitos adversos do vírus. Isso ficou evidente diante do grande número de infectados e, posteriormente, de mortos pela doença^8^. Infelizmente, os problemas de saúde não se limitaram às condições físicas; eles também se manifestaram de forma significativa no âmbito da saúde mental^32^. Antes da pandemia, a saúde mental dos idosos já era um problema de saúde de grande relevância. Estudos anteriores indicaram que quase 20% desse grupo etário sofre de algum tipo de problema de saúde mental, com diferentes graus de intensidade, os quais, por sua vez, estão relacionados a problemas cognitivos. Assim, a depressão apresenta uma relação substancial com funções cognitivas como a linguagem, a memória, a atenção e a função executiva, entre outras^30^, e talvez essa característica esteja relacionada com as conclusões deste estudo.

Sem dúvida, a infodemia, fenômeno declarado pela OMS como uma pandemia comunicativa, representa um desafio significativo para os profissionais de saúde, especialmente para os profissionais de enfermagem. Estes podem desempenhar um papel crucial no combate à infodemia, fornecendo informações precisas e atualizadas aos idosos, o que os ajudará a compreender melhor a situação e a tomar decisões informadas sobre sua saúde^34^. É fundamental educar em saúde sobre como lidar com situações complexas, como a pandemia, incluindo conselhos práticos para o manejo do estresse e da ansiedade^35^. Além disso, é essencial oferecer apoio emocional aos idosos, ajudando-os a gerenciar suas emoções e a reduzir o estresse e a ansiedade diante de fenômenos sanitários complicados. Essa abordagem integral não apenas melhora o bem-estar psicológico desse grupo etário, mas também contribui para sua capacidade de enfrentar os desafios decorrentes da infodemia.

No entanto, uma das limitações deste estudo foi a data de obtenção dos dados, o que pode ter influenciado a percepção e/ou a lembrança do fenômeno. As circunstâncias e a natureza das informações disponíveis naquele momento podem ter afetado a forma como os participantes interpretaram e reagiram à infodemia e sua relação com a saúde mental. Esse fator temporal é essencial para compreender a dinâmica da percepção, a resposta emocional em situações de crise e a lembrança delas.

## Conclusão

O estudo conclui que a relação entre a exposição à informação e à saúde mental em idosos não foi linear em nível local. Portanto, uma maior exposição nem sempre implica um impacto negativo na saúde mental. No entanto, é interessante estudar as estratégias de enfrentamento que os idosos apresentam para lidar com a infodemia. Do ponto de vista da enfermagem, é necessário implementar estratégias que promovam a saúde mental para enfrentar a desinformação, tais como: o fortalecimento da alfabetização digital e sanitária, a educação contínua no uso responsável dos meios de comunicação, o fomento de habilidades de enfrentamento emocional e a criação de espaços seguros para a escuta ativa e a expressão de emoções. Além disso, é fundamental promover redes de apoio comunitárias que fortaleçam o acompanhamento social e emocional dos idosos, bem como intervenções psicoeducativas que potencializem sua autonomia, autoestima e capacidade crítica diante da informação.

## Declaração de Disponibilidade de Dados:

O conjunto de dados deste artigo está disponível na página da RLAE no repositório Mendeley Data, no link DOI: https://doi.org/10.17632/xysrhct5ny.1

# Translation: Infodemia en COVID-19 y su relación con estrés, depresión y ansiedad en personas mayores^3^

**DOI:** 10.1590/1518-8345.7769.4749

**Authors:** Varinia Alejandra Rodríguez-Campo, Sandra Verónica Valenzuela-Suazo, Ricardo Bezerra-Cavalcante, Olivia Inés Sanhueza-Alvarado, Patricia del Tránsito Jara Concha, Aldo Vera-Calzaretta, Pablo Miguel Palma-Novoa

**Affiliations:** 1 Universidad de Concepción, Facultad de Enfermería, Concepción, Chile.; 2 Universidade Federal de Juiz de Fora, Faculdade de Enfermagem, Minas Gerais, MG, Brazil.; 3 Universidad de Atacama, Departamento Kinesiología, Atacama, Chile.; 4 Universidad de Concepción, Departamento Psiquiatría, Concepción, Chile.

**Keywords:** Infodemia, Estrés Psicológico, Depresión, Ansiedad, Anciano, COVID-19.

## Abstract

**Objetivo:**

analizar el fenómeno de infodemia en COVID-19 y su relación con estrés, depresión y ansiedad en personas mayores.

**Método:**

estudio cuantitativo, correlacional, transversal. Muestra no probabilística de 195 personas mayores, quienes respondieron cuestionario para: Infodemia, Escala de Estrés Percibido, Escala Depresión Geriátrica Yesavage, Escala de Ansiedad Generalizada, a través de encuesta telefónica y/o vía *online* autoadministrada. Previa aceptación de consentimiento informado. Se usó estadística descriptiva e inferencial para el análisis de datos. Estudio fue aprobado por Comité Ético.

**Resultados:**

la edad promedio fue de 69 ± 6,2 años, 58% fueron mujeres. El promedio de horas diarias destinadas a información fue de 5±2,5, siendo mayor en hombres, sin diferencias significativas entre sexos. Las horas de infodemia se relacionaron inversamente proporcional al padecimiento de salud mental, es así como a mayores horas de información, menor el nivel de estrés, depresión y ansiedad.

**Conclusión:**

infodemia no necesariamente tiene un impacto negativo en la salud mental de las personas mayores. Desde enfermería, se vuelve esencial fortalecer el pensamiento crítico, la alfabetización digital, promover redes de apoyo y espacios seguros que potencien la autonomía y afrontamiento ante la desinformación.

## Destacados

**(1)** La televisión fue el medio de comunicación más usado por las personas mayores chilenas. **(2)** 5 horas diarias promedio de información de COVID-19 fueron consumidas por las personas mayores. **(3)** Número de muertos e infectados generaron miedo y ansiedad en las personas mayores. **(4)** Muchas personas mayores presentaron estrés, depresión y ansiedad durante la pandemia. **(5)** Número de horas de información fue inversamente proporcional al nivel de estrés, depresión y ansiedad.

## Introducción

La humanidad siempre ha estado expuesta a diversas epidemias. Sin duda la ocasionada por Sars-Cov 2 fue una de las más complejas vividas en los últimos años^1^, debido a la alta letalidad y mortalidad ocasionada por el virus en un corto tiempo^2^, cuyas características epidemiológicas permitieron su rápida propagación desde Wuhan-China al resto del mundo^3^.

Durante este tiempo, la población en su totalidad debió cambiar sus estilos de vida a fin de prevenir los efectos adversos del virus. La Organización Mundial de la Salud (OMS) llamó a optar medidas preventivas, tales como lavado de manos, uso de elementos de protección personal, distanciamiento social y confinamiento como una de las tantas medidas para frenar la transmisión del agente patógeno^4^ ^-^ ^5^, especialmente en las personas mayores. Ellas fueron el grupo etario más comprometido a causa de la alta mortalidad del COVID-19, debido a la presencia de comorbilidades, síndromes geriátricos y fragilidad del envejecimiento^6^.

El mundo está envejeciendo, un 11% de la población mundial corresponde a personas mayores. Se prevé que para la década del 2050 este grupo etario se habrá duplicado, aumentando la población octo y nonagenaria^7^, lo que conlleva un incremento de enfermedades propias de la edad como la demencia tipo Alzheimer y otras, transformándose en un grupo vulnerable a variadas enfermedades, maltratos y desastres^7^. Es por ello que los transformó en un grupo de personas altamente vulnerables^8^ ^-^ ^10^, sobre todo por las medidas restrictivas de confinamiento y el aislamiento social, las cuales se han considerado como negativas para la población mayor debido a que generan reducción de actividad física, alteración del sueño, deterioro cognitivo y problemas de salud mental como el miedo y la depresión^8^ ^,^ ^11^.

Además, durante este periodo pandémico, millones de hogares se inundaron de un gran volumen de información que fue transmitida por canales tradicionales e informáticos, comunicando a las personas acerca de las pérdidas que generó el COVID-19 y sus medidas terapéuticas^8^. Esta información, que en muchas ocasiones fue comunicada de forma errada e incluso como noticias falsas o “*fake news*”, que no cuentan con respaldo científico, generó desinformación en las personas, orientándolas en tomar malas decisiones en salud, debido a que conspiraban con las medidas de tratamiento, farmacológicas y de vacunas relacionadas con la pandemia^12^. Fue tal la magnitud de la información transmitida muchas veces sin fundamento, que la OMS la ha denominado “infodemia”, para referirse a aquella información que se divulga rápidamente y sin mayor evidencia científica^13^ ^-^ ^14^.

Si bien las medidas restrictivas fueron las necesarias para frenar la cadena de transmisión del virus, el distanciamiento social y el confinamiento aumentaron el problema de soledad y aislamiento que las personas mayores presentaban, incluso, antes de pandemia^15^, y si además durante este periodo se inundó de información alarmante del virus conllevando a sensaciones de miedo, ansiedad, incertidumbre , estrés, frustración y otras alteraciones mentales asociadas^8^ ^,^ ^15^

El envejecimiento es un proceso natural que implica una serie de cambios anatómicos, fisiológicos y psicológicos propios de esta etapa de la vida. En este contexto, la salud mental adquiere una relevancia particular, ya que, el funcionamiento cognitivo en la vejez es un proceso individual que depende de los recursos personales, el estado de salud física y las experiencias de vida acumuladas. Se ha identificado una mayor prevalencia de trastornos mentales y una alta tasa de suicidios entre las personas mayores^16^, lo que destaca la necesidad urgente de investigar esta temática y las posibles variables que puedan afectarla, como la infodemia, con el fin de desarrollar estrategias integrales para la prevención y promoción de la salud mental. En la actualidad, más del 15% de los chilenos lo constituyen personas mayores, similar al resto del mundo^17^.

Se desconoce, a nivel local, como la información recibida durante el periodo de pandemia provocó alteraciones en la salud mental de los mismos, tal como, miedo, ansiedad, estrés y depresión, por tanto, se plantea como objetivo del estudio analizar el fenómeno de infodemia y su relación con estrés, depresión y ansiedad en personas mayores.

## Método

### Tipo de estudio

Cuantitativo, no experimental, transversal y correlacional, que siguió las recomendaciones de *Strengthening the Reporting of Observational Studies in Epidemiology* (STROBE).

### Lugar

El estudio se realizó en la región del Biobío, Chile, conformada por tres provincias Concepción, Biobío y Arauco, cada una de ellas con sus respectivas comunas.

### Periodo

El reclutamiento de los participantes fue realizado durante el periodo de marzo a septiembre de 2022.

### Población

Constituida por la totalidad de personas mayores de la región del Biobío, que para el año 2021 alcanzó un total de N=261.502 personas mayores.

### Criterios de selección

Se incluyeron en el estudio personas mayores con edad igual o superior a 60 años, que cuenten con línea telefónica o correo electrónico, y que acepten participar de la investigación. Además, deben tener la capacidad de responder encuestas en línea o por teléfono, y utilizar correo electrónico y/o redes sociales. Se excluyeron aquellas personas mayores con deterioro cognitivo que les impida responder de manera autónoma o que presenten dificultades para comprender la encuesta al momento de la entrevista.

### Definición de la muestra

Muestra de tipo no probabilística fue calculada en base a fórmula, n = N.Z².p. (1-p) /Z².p. (1-p). + e². (N-1), utilizando un error de muestreo del 5% y un nivel de confianza del 95%, lo que otorga una muestra de n=195 personas.

### Variables de estudio

Incluyeron variables biosociodemográficas (edad. sexo, estado civil, situación de pareja, ingresos económicos, etc), de infodemia, medida a través del número de horas diarias de exposición a información de COVID-19 y medios de comunicación usados (televisión, radio, redes sociales), y las variables de salud mental: estrés, depresión y ansiedad. 

### Instrumentos utilizados para recolectar información

Instrumento creado por los autores para recabar antecedentes biosociodemográficos e infodemia. Para evaluar la salud mental de los participantes se utilizó: Escala de Estrés Percibido (PSS-14), Escala de Depresión Geriátrica (Yesavage) e Inventario de Ansiedad Generalizada (GAI), todos validados en Chile^18^ ^-^ ^20^.

La Escala de Estrés Percibido (PSS-14), creada por Cohen y validada en Chile por Carvajal, et al.^19^ ^,^ ^21^, consiste en 14 preguntas tipo Likert con puntajes que oscilan entre 0 y 4. La sumatoria de los puntos permite categorizar los niveles de estrés: de 0 a 14 puntos indica que nunca o casi nunca se presenta estrés; de 15 a 28 puntos, estrés ocasional; de 29 a 42 puntos, estrés frecuente; y de 43 a 56 puntos, un nivel alto de estrés. La Escala de Depresión Geriátrica Abreviada, creada por Sheikh y Yesavage^22^ y compuesta por 15 preguntas dicotómicas, categoriza los puntajes en: de 0 a 5 puntos, normal; de 6 a 10 puntos, depresión leve; y de 11 a 15 puntos, depresión grave. Por su parte, el Inventario de Ansiedad Geriátrica (GAI), creado por Pachana^23^, y compuesto por 20 preguntas dicotómicas, permite discriminar entre personas con o sin ansiedad, considerando que un puntaje igual o superior a 8 indica la presencia de ansiedad, y un puntaje igual o superior a 10 indica ansiedad generalizada.

### Recolección de los datos

Para la recolección de la muestra, se inició el proceso seleccionando al azar un número telefónico de un listado de personas mayores del Programa de Salud Integral de Personas Mayores, en la región del Biobío, Chile. Posteriormente, se empleó el método de bola de nieve para completar la muestra. Los datos fueron obtenidos por medio de encuestas telefónicas realizadas por encuestadores entrenados o de forma autoadministrada, según la preferencia de los participantes, y fueron ingresados a un formulario de *Google Forms*, asegurando la no duplicidad de respuestas. 

### Análisis estadístico

Las respuestas obtenidas a través del formulario de *Google Forms* fueron exportadas a los paquetes estadísticos JASP (versión 0.15.0.0), Jamovi (2.3.18) e IBM SPSS *Statistics* (26.0) para su análisis. Se emplearon tanto estadísticas descriptivas como inferenciales en el análisis. Previa al análisis inferencial, se llevó a cabo una prueba de normalidad sobre la variable de horas de infodemia, la cual no cumplió con los criterios de normalidad. Posteriormente, se aplicó test de Kolmogorov-Smirnov, el cual mostró valores significativos para las variables de estrés, depresión y ansiedad. Ante estos hallazgos, se optó por utilizar el estadístico Kruskal-Wallis, basando esta decisión en la necesidad de aplicar métodos no paramétricos. Para explorar más a fondo las diferencias entre los grupos, se realizó un análisis *post hoc* utilizando la prueba de Dwass-Steel-Critchlow-Fligner (DSCF). Esta prueba es especialmente útil para comparar múltiples grupos en situaciones donde se han identificado diferencias significativas en un análisis general, permitiendo determinar específicamente cuáles grupos presentan diferencias estadísticamente significativas entre sí. El conjunto de datos que respalda los resultados de este estudio está disponible en Mendeley Data, V1, en el enlace https://doi.org/10.17632/xysrhct5ny.1.

### Consideraciones éticas

Este estudio se sustentó en los ocho requisitos de Ezequiel Emanuel, basados en las normas del Consejo de Organizaciones Internacionales de Ciencias Médicas (CIOMS) y la Declaración de Helsinki^24^. Los participantes debieron proporcionar consentimiento informado, en el cual el investigador garantizó la confidencialidad de las respuestas, así como, la mención de riesgos y beneficios asociados al estudio. La aceptación del consentimiento se registró mediante confirmación en la encuesta *web* y, en el caso de las encuestas telefónicas, se grabó la lectura del consentimiento y su aceptación, asegurando el almacenamiento en una memoria externa exclusiva para el estudio, en conformidad con la legislación chilena aplicable a investigaciones con seres humanos. El documento final, firmado por el investigador principal, fue enviado a cada participante a su correo electrónico o red social autorizada. Este estudio fue aprobado por el Comité de Ética de la Facultad de Enfermería, el Comité de Ética de la Vicerrectoría de Investigación y Desarrollo de la Universidad de Concepción, y el Comité Ético acreditado del Servicio de Salud Concepción, con código CEC-SSC:21-08-44 del año 2021.

## Resultados

El análisis descriptivo, que se presentan en Tabla 1, devela que la muestra estudiada presentó una edad promedio de 69 años, con una desviación estándar de ± 6,2 años, compuesta principalmente por mujeres, la mayoría casados y un bajo porcentaje vive en soledad. Es importante destacar que la mayoría de los participantes se encuentran jubilados y un 65% recibe un ingreso mensual de 300 mil pesos chilenos (equivalente a aproximadamente 300 US$).

**Tabla 1:** Perfil bio-sociodemográfico de personas mayores del estudio. Región del Biobío, Chile, 2022

Variables	Categorías	Fr*	%^†^
Sexo	Mujer	113	58%
	Hombre	82	42%
Estado civil	Casado/a	90	46,1%
	Viudo/a	52	26,7%
	Soltero/a	18	9,2%
	Convivencia	18	9,2%
	Separado/a	10	5,1%
	Divorciado/a	7	3,6%
Fuente de ingreso	Jubilación	175	89,7%
	Empleado/a	15	7,7%
	Beneficio Gubernamental	1	1%
Ingreso mensual	< 300 US$^‡^	127	65,1%
	301-500 US$^‡^	37	18,9%
	501-850 US$^‡^	15	7,8%
	851-1500 US$^‡^	3	1,5%
	> 1501 US$^‡^	3	1,5%
	No responde	10	5,1%
Cambio de ingresos por pandemia	Disminuye	32	16,4%
	Aumenta	31	15,8%
	No cambia	121	62%
	No responde	11	5,6%

*FR = *Fr=Frecuencia; ^†^% = Porcentaje; ^‡^Dólar estadounidense = 917 pesos chilenos el 30/09/2022

De las personas mayores estudiadas, 96% declararon haber estado expuestas a la información emitida por la televisión, 69% a redes sociales y 68% a radio. En cuanto a la afectación generada por la información emitida en pandemia, las personas mayores reportaron en un 75% sentir un impacto tanto físico y psicológico por la información emitida por televisión, 49% por la información pronunciada por la radio y 52 % se sintió afectado por la viralización de contenido en redes sociales, principalmente *Facebook* y *WhatsApp*. Entre los tipos de información recibida, la que más generó miedo entre los participantes fue la relacionada con el número de muertos e infectados, siendo particularmente alarmantes las cifras difundidas por la televisión, declarado por el 63% de las personas mayores.

El tiempo total de exposición a la información se calculó sumando las horas dedicadas a la televisión, la radio y las redes sociales, obteniendo un promedio de 5,0 horas (± 2,5 horas) diarias, con un rango que varió entre 0 y 12 horas. Al desglosar estos datos por sexo, se observó que los hombres recibieron información durante un promedio de 5,2 horas (± 2,1 horas), mientras que las mujeres lo hicieron durante 4,9 horas (± 2,7 horas), lo que evidencia una diferencia de 0,3 horas. Sin embargo, esta diferencia no fue estadísticamente significativa, con un valor de p = 0.204, lo que sugiere que el tiempo de exposición a la información no difiere de manera relevante entre ambos sexos. 

En la Tabla 2, se resumen los niveles de estrés, depresión y ansiedad, experimentados por las personas mayores participantes con sus respectivas horas diarias dedicadas a la recepción de información sobre la COVID-19 durante la pandemia. Para cada uno de los niveles mencionados, se indican las horas promedio que los participantes destinaron a la información relacionada con la pandemia.

**Tabla 2:** Horas diarias de exposición a información de COVID-19 según nivel de estrés, depresión y ansiedad en personas mayores. Región del Biobío, Chile, 2022

Horas de información						
Escalas	Nivel	Rango*	Fr^†^	%^‡^	Media^§^	DE^||^
Estrés percibido (PSS-14)	No detectado	0-14	67	34%	5,5	2,4
	De vez en cuando	15-28	114	58%	5,0	2,4
	A menudo	29-42	14	7%	3,0	2,5
	Muy estresado	43-56	0	0%	0	0
Depresión (Yesavage)	Normal	0-5	143	73%	5,4	2,5
	Depresión leve	06-10	35	18%	4,6	2,0
	Depresión grave	11-15	17	9%	3,5	2,3
Inventario Ansiedad Generalizada (GAI)	No detectada	0-7	148	76%	5,5	2,4
	Posible ansiedad	08-09	10	5%	4,6	2
	Ansiedad generalizada	10-20	37	19%	3,5	2,4

*Rango = Rango puntaje de escala; ^†^Fr = Frecuencia; ^‡^% = Porcentaje; ^§^Media = Promedio; ^||^DE = Desviación estándar

En la Tabla 3, se resumen los resultados de test de Kruskall-Wallis para las variables horas de infodemia y presencia de estrés, depresión y ansiedad en personas mayores.

Los resultados revelan la existencia de diferencias significativas entre las horas de información recibida y los distintos padecimientos de salud mental. Los hallazgos de este análisis *post hoc* se resumen en la Tabla 4.

**Tabla 3:** Relación entre horas de información de COVID-19 y la presencia de alteraciones de salud mental en personas mayores. Región del Biobío, Chile, 2022

Variables	χ²*	df^†^	p^‡^	ε²^§^
Horas de información/Estrés percibido	12,2	2	0,002^||^	0,061
Horas de información/Depresión	12,4	2	0,002^||^	0,062
Horas de información/Ansiedad	20,7	2	<,001^||^	0,104

*χ² **=** Estadístico Chi-cuadrado; ^†^df = Grados de libertad; ^‡^p = Nivel de significancia; ^§^ **ε²**= Tamaño del efecto; ^||^Significancia <0,01

**Tabla 4:** Resultados Steel-Dwass-Critchlow-Fligner para horas de infodemia, estrés, depresión y ansiedad de las personas mayores. Región del Biobío, Chile, 2022

Escalas	Gr 1*	Gr 2^†^	W^‡^	p^§^
Nivel de estrés	No detectado	De vez en cuando	-1,55	0,516
	No detectado	A menudo	-4,86	0,002^ǁ^
	De vez en cuando	A menudo	-4,29	0,007^¶^
	Normal	Leve	-2,61	0,154
Nivel depresión	Normal	Grave	-4,50	0,004^ǁ^
	Leve	Grave	-2,72	0,133
	No detectada	Posible ansiedad	-1,55	0,516
Nivel ansiedad	No detectada	Ansiedad generalizada	-6,36	<,001^¶^
	Posible ansiedad	Ansiedad generalizada	-2,13	0,287

*Gr 1 = Grupo 1; ^†^Gr 2 = Grupo 2; ^‡^W = Comparación de grupos; ^§^p = Nivel significancia; ^ǁ^Significancia p<0,05; ^¶^Significancia p< 0,01

Al observar la Tabla 4 se puede evidenciar que existen diferencias significativas entre los grupos de los distintos niveles de estrés, depresión y ansiedad estudiados. 

Al analizar y comparar los resultados presentados en las Tablas 1 y 3, se puede visibilizar que, en la escala de estrés, el grupo que no presentó estrés tuvo una exposición promedio a la información de dos horas y media más en comparación con el grupo que experimentó estrés “a menudo”. Una situación similar se observa entre los grupos “de vez en cuando” y “a menudo” en la misma escala. 

En lo que respecta a la escala de depresión, también se identifican diferencias significativas entre los grupos, destacándose una diferencia de dos horas de exposición entre el grupo clasificado como normal y el grupo que presenta un nivel de depresión grave. De manera similar, en la escala de ansiedad generalizada, se evidencia que las personas mayores que no presentaron síntomas de ansiedad tuvieron dos horas más de exposición a la información en comparación con el grupo que mostró signos de ansiedad generalizada. Es interesante observar que los grupos que no reportaron estrés, depresión o ansiedad tuvieron, en promedio, una mayor exposición a la información en comparación con aquellos que experimentaron estos padecimientos con mayor frecuencia.

## Discusión

Este estudio puso de manifiesto una relación entre la infodemia y la salud mental en la población de adultos mayores en Chile. Al comparar estos hallazgos con las tendencias observadas a nivel global, se destacan diferencias significativas en relación con otros estudios internacionales. Mientras que en el contexto global la infodemia ha demostrado una asociación positiva y más consistente con los trastornos mentales analizados, en Chile, la relación parece ser menos pronunciada^25^ ^-^ ^27^. Estos resultados sugieren que probablemente existan otros factores como culturales, sociales o económicos específicos de la población chilena que podrían influir en cómo la infodemia afecta la salud mental de los adultos mayores. Esta discrepancia subraya la importancia de considerar el contexto local al evaluar el impacto de la infodemia en la salud mental y abre la puerta a futuras investigaciones que exploren las particularidades de la experiencia chilena en comparación con otras regiones del mundo.

Durante la pandemia, las personas mayores se vieron afectadas por el aislamiento y distanciamiento social, que los llevó a mantenerse completamente en soledad^4^ ^,^ ^28^. Este tiempo favoreció otorgar tiempo para la información, la cual fue consumida preferentemente por medios tradicionales, según lo reportado por las personas mayores de este estudio. La soledad impuesta por el aislamiento y la falta de comunicación imperantes en este periodo da cabida a prestar atención a las innumerables horas de comunicación de la pandemia. 

A diferencia de otros estudios internacionales, en Chile, las personas mayores mostraron que un mayor número de horas de exposición a la información se asoció con una menor incidencia de alteraciones en la salud mental. Este resultado, sin duda, invita a los autores a reflexionar sobre el fenómeno estudiado. La aparente paradoja, en comparación con los hallazgos internacionales, suscita el debate sobre si aquellos individuos que experimentaron síntomas de estrés, depresión y ansiedad podrían haber sido más sensibles o vulnerables a la información recibida. Esto resulta plausible, pues se ha demostrado que a altos niveles de estrés, depresión y ansiedad disminuye la capacidad para procesar y retener información, que, además estas alteraciones mentales pueden afectar negativamente la función cognitiva, lo que puede dificultar la capacidad de concentración y retención de información^17^ ^,^ ^29^ ^-^ ^30^. Por otra parte, aquellos que no experimentaron síntomas de estrés, depresión o ansiedad pueden haber estado más capacitados para manejar y procesar la información, lo que les permitió mantener un nivel de exposición más alto sin verse afectados negativamente por la información recibida.

Así mismo, es importante considerar la relevancia de la televisión, que, al ser una fuente de información masiva y accesible en Chile para los sujetos estudiados, pudo haber proporcionado contenido creíble y útil durante la pandemia, a diferencia de estudios internacionales en donde el mayor consumo de contenido se efectuó por redes sociales^26^. En este sentido, la televisión podría convertirse en un medio preventivo, ayudando a las personas mayores a estar informadas de manera adecuada y a gestionar mejor sus emociones frente a nuevos eventos críticos. Así, la calidad y la credibilidad de la información recibida desempeñan un papel crucial en la forma en que los individuos perciben y responden a los desafíos de salud mental.

Es importante destacar que a pesar del efecto positivo que se observó en este grupo de personas mayores en relación con la infodemia, este grupo etario experimentó miedo y ansiedad frente a la información sobre el número de muertos y contagiados durante este período. Muchos participantes manifestaron afecciones físicas y psicológicas, lo que es consistente con hallazgos de otros estudios internacionales^31^. Esta contradicción sugiere que, aunque la exposición a la información puede haber tenido efectos protectores en algunos aspectos, el impacto emocional negativo asociado con cifras alarmantes puede haber generado una respuesta de miedo y ansiedad significativa en la población mayor.

Sin duda, durante el periodo de la pandemia de COVID-19, las personas mayores se convirtieron en un grupo altamente vulnerable a los efectos adversos del virus. Esto se hizo evidente ante el gran número de contagiados y, posteriormente, de fallecidos a causa de la enfermedad^8^. Lamentablemente, los problemas de salud no se limitaron a las afecciones físicas; también se manifestaron de manera significativa en el ámbito de la salud mental^32^. Antes de la pandemia, la salud mental de las personas mayores ya era un problema sanitario de gran relevancia. Estudios previos han indicado que casi un 20% de este grupo etario sufre algún tipo de problema de salud mental^29^, con diferentes grados de intensidad, los cuales a su vez están relacionados con problemas de cognición^33^. Así, la depresión presenta una relación sustancial con funciones cognitivas como el lenguaje, la memoria, la atención y la función ejecutiva, entre otras^30^ y quizás esta característica se vincule con los hallazgos de este estudio.

Sin duda, la infodemia, fenómeno declarado por la OMS como una pandemia comunicativa, representa un reto significativo para el personal sanitario, y especialmente para los profesionales de enfermería. Estos pueden desempeñar un papel crucial al abordar la infodemia, proporcionando información precisa y actualizada a las personas mayores, lo que les ayudará a entender mejor la situación y a tomar decisiones informadas sobre su salud^34^. Es fundamental educar en salud sobre cómo manejar situaciones complejas, como la pandemia, incluyendo consejos prácticos para el manejo del estrés y la ansiedad^35^. Además, es esencial ofrecer apoyo emocional a las personas mayores, ayudándolas a gestionar sus emociones y a reducir su estrés y ansiedad frente a fenómenos sanitarios complicados. Este enfoque integral no solo mejora el bienestar psicológico de este grupo etario, sino que también contribuye a su capacidad para enfrentar los desafíos derivados de la infodemia.

Sin embargo, una de las limitaciones de este estudio fue la fecha de obtención de los datos, lo que podría haber influido en la percepción y/o recuerdo del fenómeno. Las circunstancias y la naturaleza de la información disponible en ese momento pueden haber afectado cómo los participantes interpretaron y reaccionaron ante la infodemia y su relación con la salud mental. Este factor temporal es esencial para entender la dinámica de la percepción, la respuesta emocional en situaciones de crisis y el recuerdo de las mismas.

## Conclusión

El estudio concluye que la relación entre la exposición a la información y la salud mental en adultos mayores no fue lineal a nivel local. Por lo tanto, más exposición no siempre implica un impacto negativo en la salud mental, sin embargo, resulta interesante estudiar las estrategias de afrontamiento que presentan las personas mayores para hacer frente a infodemia. Desde la enfermería, se hace necesario implementar estrategias promotoras de la salud mental para hacer frente a la desinformación como: el fortalecimiento de la alfabetización digital y sanitaria, la educación continua en el uso responsable de medios de comunicación, el fomento de habilidades de afrontamiento emocional, y la creación de espacios seguros para la escucha activa y la expresión de emociones. Además, es fundamental promover redes de apoyo comunitarias que fortalezcan el acompañamiento social y emocional de los adultos mayores, así como intervenciones psicoeducativas que potencien su autonomía, autoestima y capacidad crítica frente a la información.

## Declaración de Disponibilidad de Datos:

El conjunto de datos de este artículo está disponible en la página de la RLAE en el repositorio Mendeley Data, en el enlace DOI: https://doi.org/10.17632/xysrhct5ny.1
